# Detecting Differential Item Functioning in Multidimensional Graded Response Models With Recursive Partitioning

**DOI:** 10.1177/01466216241238743

**Published:** 2024-03-13

**Authors:** Franz Classe, Christoph Kern

**Affiliations:** 1 39054 Deutsches Jugendinstitut, Munchen, Germany; 2 9183 Ludwig-Maximilians-University of Munich, Munchen, Germany

**Keywords:** differential item functioning, multidimensional item response theory, graded response model, categorical analysis, surveys, algorithmic modeling, machine learning

## Abstract

Differential item functioning (DIF) is a common challenge when examining latent traits in large scale surveys. In recent work, methods from the field of machine learning such as model-based recursive partitioning have been proposed to identify subgroups with DIF when little theoretical guidance and many potential subgroups are available. On this basis, we propose and compare recursive partitioning techniques for detecting DIF with a focus on measurement models with multiple latent variables and ordinal response data. We implement tree-based approaches for identifying subgroups that contribute to DIF in multidimensional latent variable modeling and propose a robust, yet scalable extension, inspired by random forests. The proposed techniques are applied and compared with simulations. We show that the proposed methods are able to efficiently detect DIF and allow to extract decision rules that lead to subgroups with well fitting models.

## Introduction

Multi-item batteries are frequently used in social scientific surveys to examine latent traits. Examples include the measurement of creativity (Jauk et al., 2014), social anxiety (Prenoveau et al., 2011), and personality disorders (Drislane & Patrick, 2017). Some traits, such as self-leadership (Furtner et al., 2015), may include multiple dimensions and can involve complex (i.e., multidimensional) measurement structures. If these latent traits are to be meaningfully used for substantive analyses, one must assume measurement invariance. This requires that the association between items of the questionnaire and latent traits of individuals do not depend on group membership. However, especially in the context of large scale surveys, the measurement invariance assumption rarely holds because of the heterogeneous nature of survey samples (Van De Schoot et al., 2015). Furthermore, a researcher can rarely identify and control all factors that jeopardize this assumption.

Measurement non-invariance is also referred to as *differential item functioning* (DIF). If group differences are found in latent factors measured by a survey questionnaire, it cannot be ruled out that this effect is only an artifact due to unnoticed DIF. That is, if DIF remains undetected, group differences can be misinterpreted. The common methods used to test for DIF usually require pre-specification of the subgroups in which DIF is assumed (Hambleton et al., 1991, p. 110). The decision which subgroups to consider for assumed DIF is often driven by theoretical priors, strong convention and biases (see Brand et al., 2019). This lets many potential relevant subgroups undetected if they do not reflect the researcher’s assumptions. Therefore, more flexible, data-driven approaches can complement traditional approaches for detecting DIF.

By using data-driven, algorithmic approaches, it is possible to detect subgroups with DIF when little theoretical guidance on the relevant subgroups is available. This strand of research includes the work of Vaughn and Wang (2010) and Schauberger and Tutz (2016), who propose data-driven methods for detecting DIF for single dichotomous items in tests or questionnaires. A particularly promising method to algorithmically account for heterogeneity is *model-based recursive partitioning* (MOB), which embeds model estimation and subgroup detection in one methodological framework (Zeileis et al., 2008). In this case, the researcher only needs to specify a set of partitioning variables along with the statistical model, which are then used to iteratively search for relevant subgroups. Tutz and Berger (2016) as well as Strobl et al. (2015) present the usage of MOB for detecting DIF in the Rasch model. Komboz et al. (2018) propose a MOB-based approach for the Partial Credit Model, called *PCM Tree*, in which a single latent variable that may be susceptible to DIF is assumed. Similar in spirit, *structural equation model tree* (SEMTree) approaches have been proposed to detect homogeneous subgroups in latent variable modeling via recursive partitioning (Arnold et al., 2021; Brandmaier et al., 2013). However, there is little guidance on how recursive partitioning may be best utilized for multidimensional measurement models with ordinal response variables.

In this study, we propose and compare recursive partitioning techniques for detecting DIF with a focus on measurement models with multiple latent variables. In terms of the response variables, we consider ordinal scales, for example, Likert or ratings scales, that are often used in social scientific applications. Such models may be referred to as *multidimensional graded response* (MGR) models. Table 1 gives an overview of the methods considered in this paper. Starting from PCM Tree, we will demonstrate that a direct analogue for graded response models using full information estimation (MML) is hardly feasible to use in practice due to its high computational costs. We therefore propose a MOB for MGR approach that eases computational burden in the multidimensional setting by focusing on limited information estimation (ML, WLS). Furthermore, we compare different algorithmic approaches provided by the partykit and the semtree packages.

**Table 1.** Comparison of tree-based methods for detecting DIF in MGR models.

Method	Estimation	Multiple latent variables?	Complex models^*^?	Computa- tionally practical?	Robustified approach?	Uncom- promised model assumptions?
PCM Tree^a^	CML (FIML)	✗	✗	✓	✗	✓
Partykit^b^	MML (FIML)^c^	✓	✗	✗	✗	✓
	ML, WLS (LIML)	✓	✓	✓	✗	✗
Naive semtree^d^	ML, WLS (LIML)	✓	✓	✗	✗	✗
Score-guided semtree^e^	ML, WLS (LIML)	✓	✓	✓	✗	✗
Partykit forest	ML, WLS (LIML)	✓	✓	✗	✓	✗
Score-guided semtree forest	ML, WLS (LIML)	✓	✓	✓	✓	✗

^*^Multivariate models with correlated latent variables or hierarchical structure.

^a^Proposed by Komboz et al. (2018).

^b^proposed by Zeileis et al. (2008).

^c^following Schneider et al. (2021).

^d^proposed by Brandmaier et al. (2013).

^e^proposed by Arnold et al. (2021).

In addition, we address the instability issues of single tree approaches when modeling DIF. Due to MOB’s hierarchical nature, small changes in the data can severely affect which subgroups are eventually identified in the splitting process (Brandmaier et al., 2016). While PCM Tree as well as the partykit and semtree approaches are susceptible to such changes, a random forest-like extension to MOB for MGR models, is analyzed that allows to robustly identify subgroups with DIF in multidimensional latent variable models.

We test and compare the outlined methods in simulations. Multiple simulation scenarios are considered that vary in the complexity of the partitioning task. The simulation results show that the proposed methods are able to correctly retrieve subgroups with distinct sets of model parameters. While partykit and semtree correctly identify subgroups in settings with clean partitioning structures, their multi-tree extensions are able to retrieve complex groups that could not have been recovered by a single decision tree. Nonetheless, computation time varies considerably across all considered methods.

## Methodology

### Methodological Background

Stochastic models which specify the relationship between single items with a limited amount of response categories and a continuous latent variable are consolidated under the term *item response theory* (IRT). Usually, in IRT models, a latent variable represents the ability of the respondent. This ability is assumed to underlie their response behavior (Steyer & Eid, 2013). In the following, we refer to this latent variable as *ξ*. Let the graded response to item *i* be denoted by the response variable *Y*_*i*_. In IRT models for ordered response variables, as opposed to dichotomous response variables, *ξ* is measured by a number of items *i* = 1, …, *m*, to which the respondent answers by choosing one of the ordered response categories *k*_*i*_ = 0, …, *l*_*i*_. The most widely applied IRT framework for items with a small amount of ordered response categories is the *graded response model* (GRM) (Samejima, 1969). Furthermore, in a multidimensional IRT framework (also referred to as MIRT, see Forero and Maydeu-Olivares (2009)) a response variable *Y*_*i*_ may be linked to more than one latent variable. In the following, we refer to the *multidimensional GRM* as MGR model. For an MGR model, ***ξ*** is a *p* × 1 vector containing all latent variables *ξ*_*g*_ *∀ g* = 1, …, *p*.

The fact that the latent variables are measured by graded responses on items means that the probability of answering in a category smaller or equal to a certain ordered category *k*_*i*_ depends on the (multidimensional) distribution of the latent variables. In the MGR model, this relationship is defined by the cumulative category response function, that is the ***ξ***-conditional probability function

(1) $$P\left(Y_{i}\geq k_{i}|\xi \right)=\Phi \left(\beta_{i}^{\prime }\xi -\alpha_{ik}\right).$$

The link function Φ is the distribution function of the standard normal distribution. The threshold parameter *α*_*ik*_ is the location on the underlying latent variable space where *P*(*Y*_*i*_ ≥ *k*_*i*_ | ***ξ***) = 0.5. The threshold parameters are, per definition, ordered in size, so that *α*_*i*1_ < *α*_*i*2_ < … < *α*_*il*_. Note that for every item *i* there is one threshold parameter *α*_*ik*_ less than the total number of ordered categories *l*_*i*_ within item *i*. The discrimination parameters *β*_*ig*_, that make up the *p* × 1 vector ***β***_*i*_, can be interpreted as the slope parameters of the multidimensional probability function *P*(*Y*_*i*_ ≥ *k*_*i*_ | ***ξ***) for all categories *k*_*i*_ = 0, …, *l*_*i*_ of item *i*. Because IRT parameters specify the relation between items and latent variables, we will refer to the MGR model parameters as item parameters, which form the item parameter vector, that is

(2) $$\vartheta =\left\{\alpha_{11},\ldots ,\alpha_{ml},\beta_{11},\ldots ,\beta_{mp},Var\left(\xi_{1}\right),\ldots ,Var\left(\xi_{p}\right),Cov\left(\xi_{1},\xi_{2}\right),\ldots ,Cov\left(\xi_{p-1},\xi_{p}\right)\right\}.$$

Note that Var(*ξ*_*p*_) is fixed to 1 if *β*_1*p*_ is freely estimated (and vice versa). Also, estimating covariances between latent variables has an impact on the estimation of item threshold and discrimination parameters. We therefore consider latent variable variances and covariances as item parameters.

In IRT models, *differential item functioning* (DIF) occurs if an item parameter depends on covariates of ***Y***, that is a *m* × 1 vector of observed response variables. Such covariates can take the form of characteristics of the individuals who respond to the items. Different scores on these covariates classify different subgroups in the population. The item parameters for each of these subgroups may differ. The difficulty of an item may, for example, depend on ethnicity, education, or gender. Differential item functioning means that the item parameter vector ***ϑ*** depends on the covariate vector ***Z***. It does not necessarily mean that the latent variable vector ***ξ*** also depends on ***Z***. This implies that DIF is present when the probability of responding to an item is different for two individuals with the same ability, only because of their group membership.

In practice, DIF can be very problematic because the number of relevant covariates may be large. Also, there is an even greater amount of possible values or value ranges of these covariates for which the item parameters might differ. In addition, complex interactions within the covariate vector ***Z*** are possible so that subgroups may only be detected by considering several covariates jointly. If DIF remains undetected, group differences with respect to the latent variables can be misinterpreted (Komboz et al., 2018).

Usually, the hypothesis ***ϑ***_1_ ≠ ***ϑ***_2_, where *h* = 2 stands for a focal subgroup and *h* = 1 stands for a reference group, can be tested empirically. Let’s assume that, in this exemplary case, the subgroups that are tested for DIF are split at the median on the metric covariate *Z*_1_. In this situation, the *Likelihood Ratio* (LR) test can be applied to test if an *augmented* model, where all item parameters are allowed to vary across the two groups, outperforms a *template* model, in which all item parameters are constrained to be equal across the reference and the focal group (Bulut & Suh, 2017). If this is the case, the researcher must assume DIF for these two groups.

Turning to model parameter estimation, social scientists often use *confirmatory factor analysis* (CFA) to operationalize and estimate latent variable models with Likert-scale items (Li, 2016). In a classic CFA model, the observed items are assumed to be measured on a continuous (metric) scale. The basic factor analytic model with intercepts is

(3) $$Y=\pi +\beta^{\prime }\xi +\epsilon ,$$

where ***ϵ*** is the *m* × 1 vector of residual variables and ***π*** is the *m* × 1 vector of intercepts representing the expected values of *Y*_*i*_ *∀ i* = 1, …, *m*, when the values of ***ξ*** are zero (Jöreskog, 1969). Note that model fit is not affected by the estimation of intercepts. In the factor analytic framework, the model parameter vector is

(4) $$\theta =\left\{\pi_{1},\ldots ,\pi_{m},\beta_{11},\ldots ,\beta_{mp},Var\left(\xi_{1}\right),\ldots ,Var\left(\xi_{p}\right),Cov\left(\xi_{1},\xi_{2}\right),\ldots ,Cov\left(\xi_{p-1},\xi_{p}\right),Var\left(\epsilon_{1}\right),\ldots ,Var\left(\epsilon_{m}\right)\right\}.$$

The CFA approach can also be used to estimate MGR model parameters. For this, a continuous, normally distributed latent response variable $Y_{i}^{*}$ is assumed to underlie each observed response variable *Y*_*i*_ for item *i* (Muthén, 1984). In the factor analytic approach for ordinal items, the latent response variable $Y_{i}^{*}$ of item *i* is related to the observed categorical response variable *Y*_*i*_ via a threshold relation, that is

(5) $$Y_{i}=k_{i}\;\text{if}\;\alpha_{ik}<y_{i}^{*}<\alpha_{i\left(k+1\right)}.$$

It is assumed that a respondent chooses a response category *k*_*i*_ when the respondent’s latent response value $y_{i}^{*}$ lies between thresholds *α*_*ik*_ and *α*_*i*(*k*+1)_.

Parameter estimation in the factor analytic framework for metric items is usually done with the maximum likelihood (ML) estimator (Jöreskog, 1969). The use of ML estimation in SEM requires the assumption that the observed variables follow a multivariate normal distribution (Li, 2016). Note that this assumption rarely holds for ordinal items. In the factor analytic framework for metric items only univariate and bivariate information is used for parameter estimation. For this, the objective function *F*_*ML*_ is minimized, that is

(6) $$F_{ML}\left(\theta \right)=\ln\left|\Sigma \left(\theta \right)\right|+tr\left(S\Sigma^{-1}\left(\theta \right)\right)-\;\ln\left|S\right|-m,$$

where **Σ**(***θ***) is the model implied covariance matrix and ***S*** is the sample covariance matrix (Jöreskog, 1969). This approach for parameter estimation is thus called *limited information approach* (LIML) and is computationally more efficient than the *full information approach* (FIML, see SupplementalMaterial S3).

Calculating the log-likelihood function for every single individual *j* in the sample, that is

(7) $$\begin{array}{l} \ln L\left(y_{j},\theta \right)=-\frac{1}{2}\left\{\ln\left|\Sigma {\left(\theta \right)}_{j}\right|+{\left(y_{j}-\pi_{j}\right)}^{T}\Sigma {\left(\theta \right)}_{j}^{-1}\left(y_{j}-\pi_{j}\right)\right\},\\ \forall j=1,\ldots ,n, \end{array}.$$

where ***π***_*j*_ denotes the subvector of the model-implied mean vector and **Σ**(***θ***)_*j*_ denotes the submatrix of the model-implied covariance matrix with respect to ***y***_*j*_. Summing the results of equation (7) across the whole sample and maximizing the results yields asymptotically equivalent parameter estimates to limited information maximum likelihood estimation (Lee & Shi, 2021). The derivative of equation (7) can easily be derived from a model that has been fitted with *F*_*ML*_. This derivative is also referred to as the score function and is particularly important for parameter instability testing.

It is also possible to use the limited information approach to parameter estimation for factor analysis with ordinal items. As mentioned above, normal distribution of the observed response variables cannot be assumed in this case. However, through the use of an asymptotically distribution free *weighted least squares* (WLS) estimator, normal distribution of the observed response variables need not be assumed. Prior to parameter estimation, the thresholds that define the relation of Y* to ***Y*** (see equation (5)) are estimated through bivariate contingency tables. Additionally, bivariate polychoric correlations are estimated in this step (Muthén, 1984). A polychoric correlation captures the strength of the considered linear dependence between $Y_{i}^{*}$ and $Y_{s}^{*}$ for *i* ≠ *s*. The model parameters are then estimated through minimization of the WLS fit function, that is

(8) $$F_{WLS}\left(\theta \right)={\left[\hat{\kappa }-\kappa \left(\theta \right)\right]}^{\prime }\hat{W}\left[\hat{\kappa }-\kappa \left(\theta \right)\right],$$

where **κ**(***θ***) contains the vectorized elements of the lower half of the model implied covariance matrix **Σ**(***θ***) and $\hat{\kappa }$ is a vector of corresponding polychoric correlation estimates below the diagonal of the polychoric correlation matrix ***K***. The weight matrix $\hat{W}$ is the asymptotic covariance matrix of the polychoric correlation estimates $\hat{\kappa }$ . The weight matrix is supposed to account for distributional variability among the observed variables (Li, 2016).

Both CFA and MGR models can be consolidated under the *structural equation model* (SEM) framework, as both models hypothesize about multivariate constructs by specifying relationships between observable and latent variables.

### Model Based Recursive Partitioning to Detect Differential Item Functioning

The application of tests such as the LR test to detect DIF requires a priori specification of the analyzed groups. Often though there are several numerical or categorical covariates and a large number of possible splitting points and the researcher may not have specified theoretical priors for all of the possible subgroups. Consequently, some subgroups with DIF might remain uncovered. In cases like this, recursive partitioning can be used as a data-driven method to uncover relevant groups for DIF. Recursive partitioning methods follow tree-based, algorithmic approaches (Breiman et al., 1984). In recursive partitioning, the full sample sits at the root of a decision tree. This root is considered a candidate for potential splitting into subgroups with respect to any of the covariates *Z*_*r*_ in {*Z*_1_, …, *Z*_*R*_} (also called partitioning variables). A subgroup represents a tree node, which in turn is a candidate for further splitting. The algorithm may continue splitting until certain predefined stopping criteria are met. This is usually the case when there is no more significant instability in a tree node or when the subsample becomes too small. The terminal nodes of a decision tree are also called leaves. There are several methods that can be grouped under the umbrella term *Model Based Recursive Partitioning* (MOB), which we present below.

Originally, *Structural equation model trees* (SEM Trees), as presented by Brandmaier et al. (2013), combine recursive partitioning with the LR test. The algorithm searches through all partitioning variables to find subgroups that differ with respect to the model parameters. It is implemented in the semtree package (Brandmaier et al., 2015).

With the original (or “naive”) semtree approach, the parameters in ***θ*** are first estimated jointly for the entire sample using an M-estimator (like the ML estimator, see section methodological background). Then, the augmented models for all possible split points of all partitioning variables *Z*_*r*_ in {*Z*_1_, …, *Z*_*R*_} are fitted. Note that especially if there are several (unordered) categorical and numerical partitioning variables, this means that there is a large number of augmented models to fit. However, this step is necessary to compute the log likelihood ratio for every augmented model against the template model. For every partitioning variable, the maximum log likelihood ratio is used to set the optimal split point. Then, the LR test is performed for every partitioning variable. The partitioning variable *Z*_r*_ with the smallest *p*-value in the LR test is then chosen for splitting. If none of the partitioning variables show a significant *p*-value, the partitioning process is stopped. Bonferroni adjustments may be used to account for multiple comparisons. The procedure results in a tree structure with one fitted SEM for each terminal node.

One clear advantage of the naive semtree approach, compared to the LR test, is that the researcher does not need to pre-specify the functional form between the covariates and DIF. Rather, the tree structure is learned from the data in an exploratory way (Brandmaier et al., 2013). Another advantage is the ease of interpretation of the resulting subgroups. They are directly interpretable because they are built on traceable sample splits. Thus, the advantage that no pre-specification of subgroups is necessary, as in mixture models (Rost, 1990), are combined with the advantage of the LR approach, that the resulting subgroups are interpretable with respect to covariates. However, the high computational cost of this method can make its application on large data sets and complex models unfeasible.

A similar recursive partitioning approach is provided in the partykit package by Hothorn and Zeileis (2015). In contrast to the naive semtree approach, partykit tests a fitted model in a node for parameter instability with respect to any of the partitioning variables. If there is significant parameter instability, the node is eventually split at a point on the covariate with the greatest instability into two locally optimal segments. If an M-estimator is used to fit the model, parameter instability of the fitted model with respect to a covariate can be detected through the generalized M-fluctuation test (Zeileis & Hornik, 2007). The null hypothesis of the generalized M-fluctuation test is rejected if the empirical fluctuation during parameter estimation with respect to a covariate is improbably large.

Following Stefanski and Boos (2002), an M-estimator $\hat{\theta }$ is defined as the solution to the equation

(9) $$\overset{n}{\underset{j=1}{\sum }}\psi \left(y_{j},\theta \right)=0.$$

In the context of SEM, *ψ* is a (*k* × 1)-function where *k* denotes the number of parameters estimated in a SEM model. The estimator $\hat{\theta }$ is the solution that minimizes the model’s objective function (e.g., *F*_*ML*_ or *F*_*WLS*_, see equation (6) and (8)). For ML estimation, $\psi \left(y_{j},\hat{\theta }\right)$ is the derivative function of the individual contributions to the model’s log likelihood with respect to the parameter vector (see equation (7)). For $\hat{\theta }$ , the derivatives add up to zero across all individuals in the sample. For *k* parameters in the latent variable model, the derivative function is

(10) $$\psi \left(y_{j},\hat{\theta }\right)=\left(\frac{\partial \ln L\left(y_{j},\hat{\theta }\right)}{\partial {\hat{\theta }}_{1}},\ldots ,\frac{\partial \ln L\left(y_{j},\hat{\theta }\right)}{\partial {\hat{\theta }}_{k}}\right),\forall j=1,\ldots ,n.$$

The generalized M-fluctuation test uses the function $\psi \left(y_{j},\hat{\theta }\right)$ to derive tests statistics that capture the empirical fluctuation process across all parameter estimates in $\hat{\theta }$ . For this, different kinds of test statistics can be used. For example, for numerical covariates, partykit uses a test statistic that is equivalent with the *maxLM* statistic from Merkle and Zeileis (2013). To assess instability with respect to categorical or ordinal covariates, different kinds of test statistics based on the sum of the scores in every category are used.

The generalized M-fluctuation test rejects the null hypothesis of “no structural change” when the empirical fluctuation process becomes exceptionally large in comparison to the fluctuation of the limiting process. This limiting process is represented by the limiting distribution which can be approximated as closed form solutions to certain functions. If closed form solutions are not possible, critical values for hypothesis testing can be simulated “on the fly” (Zeileis, 2006a). Although solutions in closed form are faster, the *p*-values can be calculated very quickly in this way. The generalized M-fluctuation test is provided in the strucchange package (Zeileis et al., 2002).

Note that the function $\psi \left(y_{j},\hat{\theta }\right)$ is easily obtained for ML estimation. As mentioned in section 2.1, from SEM models fitted with the limited information ML method, individual log-likelihood values (equation (7)) can be easily derived (see Zeileis, 2006b). However, this is not (yet) the case for SEM models fitted with the limited information WLS method. Parameter instability tests for MGR models fitted with WLS are not yet available. In this paper, we therefore do not directly apply the M-fluctuation test to models fitted with WLS.

In every node of a decision tree partykit tests for parameter instability. If there is overall parameter instability in the current node, that is, if the instability test for any of the partitioning variables falls below a prespecified significance level, the partitioning variable $Z_{r^{*}}$ that is associated with the smallest *p*-value is chosen for splitting. To find the optimal split point in a binary partykit decision tree, the segmented objective functions of two rival segmentations are compared until the optimal split point on $Z_{r^{*}}$ is found (Zeileis et al., 2008, p. 498f.). Note that this requires fitting as many models as there are possible segmentations of the partitioning variable $Z_{r^{*}}$ .

Compared to the naive semtree approach, one advantage of partykit is reduced computation time. To apply the generalized M-fluctuation test to all partitioning variables, the model needs only be fitted once. Split point selection, however, is more time consuming because the model has to be fit for all possible segmentations of the selected partitioning variable.

The idea of testing a fitted model in a node for parameter instability with respect to the partitioning variables is also used in the “score-guided” semtree approach (Arnold et al., 2021), which supersedes naive semtree. As with the partykit method, the first step of the algorithm is to select the partition variable. This is done in the same way as in partykit, through the generalized M-fluctuation test.

The key difference between partykit and score-guided semtree is that the latter performs a different procedure than partykit for selecting the split point given a selected partitioning variable. Instead of calculating the log likelihoods for all possible rival segmentations, score-guided semtree identifies which of the unique values of a partitioning variable maximizes the respective score-based test statistic (Arnold et al., 2021, p. 8). As a result, the model only needs to be fitted once at each node of the decision tree. Compared to the partykit method, score-guided semtree can further reduce computation time in the construction of the decision tree. For the generalized M-fluctuation test, both partykit and score-guided semtree use the *supLM* (or equivantly *maxLM*) test statistic for metric covariates and the *LMuo* statistic for categorical variables (see Merkle & Zeileis, 2013). Score-guided semtree uses the maxLM statistic for ordered variables (*maxLMo*) (Merkle et al., 2014). All these test statistics are implemented in the strucchange package.

A drawback of naive and score-guided semtree as well as partykit is their instability towards small changes in the data because of the hierarchical nature of the tree growing process. The position of a split point in the partition determines how the sample is split up in new nodes. The position of the split point as well as the selection of the splitting variable, however, strongly depend on the particular distribution of the data. The entire structure of the tree could be altered if one splitting variable or split point was chosen differently (Strobl et al., 2009).

### Recursive Partitioning for Multidimensional Graded Response Models

As mentioned in Section 2.2, recursive partitioning can be applied to any kind of parametric model that is fitted using an M-estimator (e.g., maximum-likelihood). Komboz et al. (2018) propose a recursive partitioning algorithm to detect DIF in the *Partial Credit Model* (PCM), called *PCM Tree*. The PCM is another model from the IRT framework. The PCM Tree algorithm includes a global test for measurement invariance. If there is significant item parameter instability with respect to any of the covariates *Z*_*r*_ in ***Z***, then the assumption of measurement invariance (no DIF) should be rejected.

In PCM Tree, only one latent variable *ξ* can be considered in the models that are associated with the tree’s nodes and thus multidimensional graded response (MGR) models cannot be handled. A direct analogue to PCM Tree for MGR models would draw on full information parameter estimation in the tree growing process (see Schneider et al., 2021). In Supplemental Material S3, however, we establish that model based recursive partitioning for MGR models using the full information approach is rarely feasible due to enormous computational costs. Thus, in order to conduct MOB for MGR models, computationally efficient approaches are needed.

We present and compare practicable methods to test and control for differential item functioning for complex survey scales and large scale survey data. Particularly, we suggest to combine the limited information approach for parameter estimation (Section 2.1) and recursive partitioning algorithms (Section 2.2) in order to efficiently compute MGR model based decision trees and to evaluate the resulting models with regard to model fit.

#### Recursive Partitioning for Multidimensional Graded Response Models: Single Tree

In this section, we introduce different ways to efficiently compute a single recursive partitioning tree for MGR models. We distinguish between the tree growing process (first step) and the terminal node model estimation process (second step). On this basis, we draw on different estimators to detect subgroups with DIF and to estimate fit indices and parameter estimates in an MGR modeling context. We present three algorithms, utilizing the semtree and the partykit packages (Section 2.2). The proposed methods are summarized schematically in Supplemental Material S1 in Algorithm 1, 2, and 3. Note that the algorithms differ with respect to the tree growing process as implied by the different packages used.

To start tree growing with the naive semtree approach, numerous models have to be fitted for which the log likelihoods are then compared with the template model. In the first step of the partykit method and the score-guided semtree method, the score function (see equation (10)) is used to build the tree structure. Usually, the MML estimation method is too computationally expensive for these approaches (see Supplemental Material S3). To efficiently calculate log-likelihoods for naive semtree and the score function for partykit and score-guided semtree, we propose to use (limited information) ML estimation in the tree growing process, that is, parameter estimates are computed by minimizing the objective function of the ML estimator (equation (6)). Thus for all three algorithms, we compromise on our assumptions about the distribution of the response variables. In the first step of the proposed recursive partitioning approaches for MGR models, information is used that is based on the assumption that the observed variables follow a continuous multivariate distribution. This may lead to problems in the tree growing process. In this study, we therefore analyze tree stability using data with simulated numeric response variables (based on a traditional CFA model) and compare the resulting trees to those grown using data with ordinal response variables (based on a MGR model).

Note that for partykit and semtree for MGR models, the M-fluctuation test uses the partial derivative of the objective function with respect to the model parameter vector ***θ*** (as opposed to the item parameter vector ***ϑ***). This means that individual contributions to the score function include individual deviations with respect to residual variances and nodes are split to minimize the interindividual variance with respect to these parameters. However, these parameters don’t exist in the original GRM. In the MGR model, DIF occurs if the item parameter vector ***ϑ*** depends on covariates of the response variables (see Section Methodological Background). Thus, strictly speaking, the partial score function with respect to the item parameter vector, $\psi \left(Y,\hat{\vartheta }\right)$ , needs to be considered for DIF detection through partykit or semtree. In Supplemental Material S3, we apply the MOB method to detect DIF with respect to the item parameter vector ***ϑ***. However, this method turned out to be nearly infeasible due to high computational costs as outlined above. The estimation is computationally expensive because multidimensional integrals have to be solved in order to minimize the objective function. Using this full information approach, however, individual contributions to the minimization of the objective function are considered and the function $\psi \left(Y,\hat{\vartheta }\right)$ is derived.

In the second step of our proposed algorithms, the parameter and model fit estimates of the models that are stored in the terminal nodes of the decision tree are calculated using the distribution free weighted least squares (WLS) estimator. Thus, for evaluation of the resulting decision tree, the model fit indices in the terminal nodes are estimated under consideration of non-normally distributed response variables and the existence of the threshold relation between the response variable vector ***Y*** and latent response variable vector Y*. Thus, parameters and standard errors are only estimated for models that fit the data within the subgroup. Along with sufficient sample size, this is very important for correctly estimating parameters and standard errors. Parameters in models in which the parameters are stable but which don’t fit the data are unlikely to be interpretable.

#### Recursive Partitioning for Multidimensional Graded Response Models: Forests

While the outlined methods allow to efficiently grow a single decision tree, this method may be slightly inaccurate because MGR model assumptions are compromised. At some splitting points in the decision tree, variable and split point selection may be different if the objective function considered all parameters and distributional assumptions of the MGR model (see also Supplemental Material S2). Also, a single decision tree can be vulnerable to small changes to the data and to the set of partitioning variables. This is a consequence of the hierarchical nature of the splitting process (Brandmaier et al., 2016; Kern et al., 2019)—the selection of one particular partitioning variable $Z_{r^{*}}$ at the root node determines the entire tree structure.

Using the computation time saving method described above, we are able to tackle the problem of unstable and potentially inaccurate trees by computing several structurally different trees and evaluating the compiled results of the tree ensemble. As the computation of a decision tree using partykit and score-guided semtree is considerably less time consuming compared to the naive semtree approach (Arnold et al., 2021) we only consider these methods (i.e., Supplemental Material S1, Algorithm 2 and 3) as base learner in the ensemble.

We are guided by the concept of random forests, a method that uses an ensemble of decision trees rather than a single one to enhance prediction performance (Breiman, 2001a). We use random split selection to grow decorrelated trees for the ensemble that are structurally different from each other. In this procedure, random selections of partitioning variables are made. The selection of partitioning variables is redrawn at every node in a decision tree. This way, we encourage that all partitioning variables are considered at least once, even if a small number of trees are computed. Another technique used in the random forest framework is bagging. If bagging is used, the tree growing algorithm is applied to a bootstrap sample drawn from the full sample at every iteration. However, we refrain from using bagging together with recursive partitioning for MGR models. We want to ensure that the parameter estimates in the subgroups that are found by the algorithm are directly replicable. This is necessary to ensure that the fit indices of the fitted models are comparable between the trees.

The steps performed to grow a forest of partykit trees or score-guided semtrees for MGR models are summarized in Supplemental Material S1 in Algorithm 4. Multiple decision trees are grown using either partykit or semtree for MGR models (see section Recursive Partitioning for MGR models: Single Tree) with random sampling of partitioning variables at each node. After multiple decision trees are grown, the fit indices of the fitted models in the terminal nodes of each decision tree are evaluated. In this step, fitted models in terminal nodes that don’t exceed a predefined cutoff criterion (*χ*^2^-test *p*-value or RMSEA cutoff) are selected. The forest outputs a list of subgroups for which the proposed MGR model holds and DIF is present.

## Simulations

### Measurement Model

We test and compare the presented recursive partitioning techniques for MGR models with simulations. For this, a multidimensional graded response model needs to be defined. In the following, the simulated data is created based on the assumptions of the *probit multistate IRT model with latent item effect variables for graded responses* (PIEG, Classe & Steyer, 2023a).

The PIEG model is a multistate model with latent item effect variables for ordinal observables. For every category of a response variable, one category-specific latent state variable *τ*_*ikt*_ for category *k* of item *i* at time point *t* is defined in the PIEG model. One reference latent state variable *η*_*t*_, which is equal to the latent state variable of the reference item *τ*_11*t*_, is assumed for every time point of measurement. The latent item effect variable *β*_*i*_ is defined as the difference between the latent state variable of the reference item and the latent state variable of another item. Thus, there are as many latent item effect variables as there are items, minus the reference item. In this model, variances and covariances of latent state variables, and latent item effect variables as well as the covariances between latent item effect variables and latent state variables are estimated. The model’s discrimination parameters are all fixed at 1. For our application, all threshold parameters are freely estimated.

To simulate data on the basis of the PIEG model, we define three reference latent state variables *η*_*t*_ and two latent item effect variables *β*_*i*_. We are thus mimicking a longitudinal setting with data collected for three time points. The proposed latent variables are derived from three items, respectively, resulting in nine five-category ordinal response variables *Y*_*it*_. The model structure is shown in Figure 1 in Supplemental Material S1. The cumulative category response function of the PIEG model is

(11) $$\begin{array}{l} P\left(Y_{it}\geq k_{i}|\eta_{t},\beta_{i}\right)=\Phi \left(\eta_{t}+\beta_{i}-\kappa_{ikt}\right),\\ \forall k=1,\ldots ,4,\forall i=2,\ldots ,3,\forall t=1,\ldots ,3. \end{array}$$

In this model, there are 36 free threshold parameters (4 for every five-category item), 10 free covariances between the latent variables, and 5 free variances of the latent variables, resulting in 51 free parameters in total.

To additionally simulate data with which ML estimation can be performed without compromising model assumptions, we define a traditional CFA model for which the response variables are numerical and follow the normal distribution. The model function is

(12) $$\begin{array}{l} Y_{it}=\pi_{it}+\eta_{t}+\beta_{i}+\epsilon_{i},\\ \forall i=2,\ldots ,3,\forall t=1,\ldots ,3. \end{array}$$

where *π*_*it*_ is an item- and time-specific intercept and *ϵ*_*i*_ is an item-specific residual variable. In this model, there are 10 free covariances between the latent variables, 5 free variances of the latent variables, 9 free intercepts and 9 free residual variances resulting in 33 free parameters in total.

For all data sets, several partitioning variables *Z*_*r*_ *∀ r* = 1, …, *R* are simulated. Different subgroups *R*_*h*_ *∀ h* = 1, …, *H* for which DIF is present may be defined as different areas on the (multidimensional) distribution of these partitioning variables.

### Simulation Setup

We create simulated data to test and compare the performance of partykit, naive and score-guided semtree for MGR models. Single decision tree approaches are applied to the first set of simulations (simulation 1) while ensemble techniques are applied to the second set of simulations (simulation 2). We conduct additional simulations to test the performance of the generalized M-fluctuation test under misspecification in Supplemental Material S2. R implementations of the proposed methods and replication materials for all simulations are provided in the following OSF repository: https://osf.io/sv35m/?view_only=6cdde2777b914322b32ca00ad567ff2b.

#### Simulation 1

**Figure 1.**
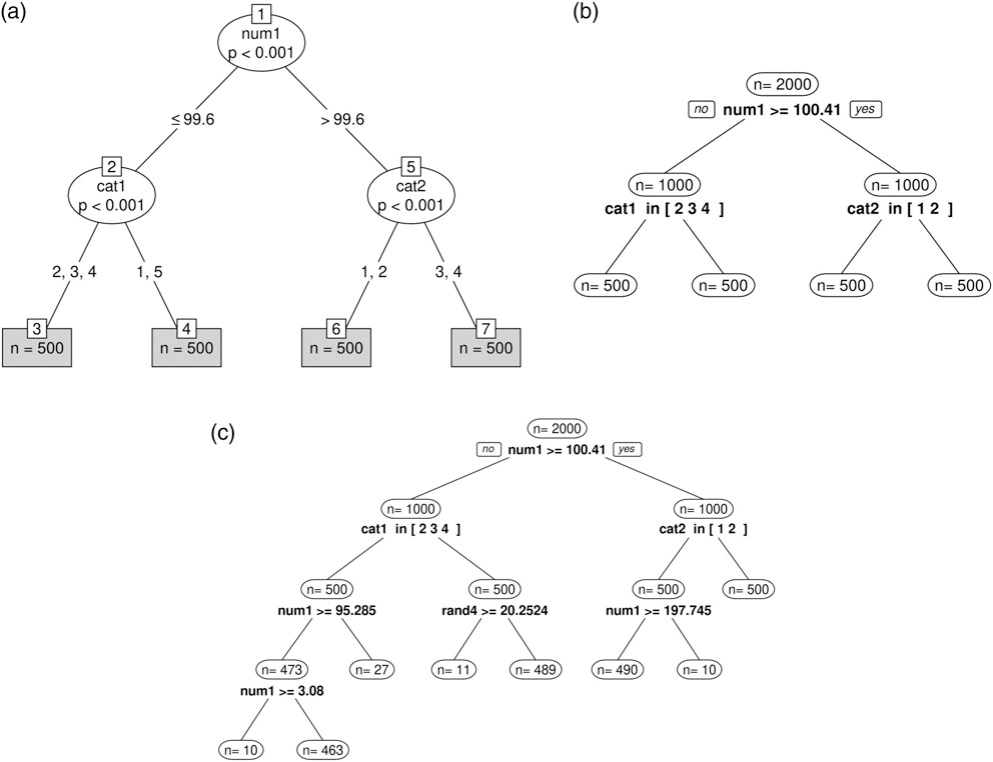
Results of single sample application of simulation 1. (a) Partykit for MGR models. (b) Score-guided semtree for MGR models. (c) Naive semtree for MGR models.

The samples of simulation 1 each consist of 2000 observations with values on 17 variables. There are no missing data points in the samples. We simulate 9 response variables in two ways: One set of samples with ordinal response variables that are based on the model function in equation (11) (see Figure 1). We also created a set of numeric samples that are based on the model function in equation (12). For each (ordinal and numeric) sample, we created two ordinal variables (cat1 and cat2) with scores on a five-point Likert Scale and one numerical variable (num1) ranging from 1 to 200. Those three variables are relevant partitioning variables. This means that they allow to distinguish between four subgroups with 500 observations in each group. Additionally, for each sample five random partitioning variables (rand1 to rand5) were simulated that do not systematically differentiate among the four subgroups. There are two numerical and three ordinal random partitioning variables.

We simulated 100 ordinal samples and 100 numeric samples. For each sample, the data for each subgroup is simulated with a different set of parameters so that the model function in equation (11) (for the ordinal data) or equation (12) (for the numerical data) is true for each subgroup, but there is DIF in the overall sample. The true group-specific parameters differ between the samples. Intercepts and threshold parameters were sampled from a normal distribution, and latent variable variances and covariances were sampled from a uniform distribution. Further details and code for replication purposes is provided in the OSF repository. For each subgroup within one single sample, the values on the relevant partitioning variables are simulated such that each subgroup is exclusive with respect to the values of the relevant partitioning variables. Additionally, the structure of the simulated sample can be broken down by a single decision tree. The subgroups are defined as

$$\begin{array}{l} R_{1}:=\left\{\left\{\text{num}1<100\right\}\cap \left\{\text{cat}1\in \left\{1,5\right\}\right\}\right\},\\ R_{2}:=\left\{\left\{\text{num}1<100\right\}\cap \left\{\text{cat}1\in \left\{2,3,4\right\}\right\}\right\},\\ R_{3}:=\left\{\left\{\text{num}1\geq 100\right\}\cap \left\{\text{cat}2\leq 2\right\}\right\},\\ R_{4}:=\left\{\left\{\text{num}1\geq 100\right\}\cap \left\{\text{cat}2\geq 3\right\}\right\}. \end{array}$$

All subgroups within one single sample fit the assumed model very well (RMSEA of 0.05 or lower for the models shown in equations (11) and (12), respectively).

We conduct the simulation analysis in two steps. In the first step, we apply partykit, naive and score-guided semtree for MGR models to one single ordinal sample of simulation 1 to test if the methods are able to detect DIF and to compare runtime results for a sample that has a clear subgroup structure. In the respective model setup, we do not impose constraints on the minimum sample size in the terminal nodes. Bonferroni adjustments are applied at every node to correct for the multiple comparisons arising from the repetition of the generalized M-fluctuation test (for partykit and score-guided semtree) or of the LR-test (for naive semtree). The number of hypothesis repeated at every node is equal to the number of partitioning variables used.

The PIEG model fit the four subsets of this sample very well (*R*_1_: RMSEA <.001,95%C.I.=.000−.034 , *R*_2_: RMSEA <.001,95%C.I.=.000−.033 , *R*_3_: RMSEA = .025, 95% *C*.*I*. = .000 − .048, *R*_4_: RMSEA = .013, 95% *C*.*I*. = .000 − .041). Through Monte Carlo simulation, Classe and Steyer (2023b) found that the quality of the parameter estimates and standard errors associated with the PIEG model are very good for sample sizes of 500, given the model fits the data. We therefore assume that recovery of the simulated subgroups, in which the models fit very well, results in accurate parameter estimation within these subgroups. The input parameters for all subgroups (*R*_1_ to *R*_4_) in this sample that are used for data generation are shown in Tables 1 and Table 2 in Supplemental Material S1.

In the second step, we apply partykit and score-guided semtree to all 100 ordinal and 100 numerical samples and analyze tree stability across simulations.

#### Simulation 2

The samples of simulation 2 each consist of 2000 observations on 18 variables. Again, there are five random partitioning variables in these samples. In addition, there are four relevant partitioning variables: cat1 (categorical), cat2 (ordinal), num1 (numerical) and dicho1 (dichotomous). The relevant partitioning variables differentiate among two (exclusive) subgroups defined as

$$\begin{array}{l} R_{1}:=\left\{\left\{\text{cat}2\geq 3\right\}\cap \left\{\text{cat}2\leq 4\right\}\cap \left\{\text{num}1\leq 50\right\}\right\},\\ R_{2}:=\left\{\left\{\text{dicho}1=0\right\}\cap \left\{\text{cat}1\in \left\{1,4,5\right\}\right\}\right\}. \end{array}$$

The subgroups *R*_1_ and *R*_2_ consist of 500 observations each (within one single sample). The data for the subgroups are simulated to fit the PIEG model well but with different sets of parameters such that DIF is present. Again, 100 ordinal samples as well as 100 numeric samples are created. The values of the simulated response variables for the remaining half of each sample of simulation 2 are random. For the ordinal samples, this means that values between 1 and 5 were randomly sampled for all response variables for all individuals that did not belong to *R*_1_ or *R*_2_. For the numerical samples, the values were randomly sampled from a uniform distribution with a minimum of −3 and a maximum of 3. Consequently, the PIEG model only holds true for subgroups *R*_1_ and *R*_2_. Additionally, the simulated subgroup structure of the sample of simulation 2 cannot be recovered by one single decision tree.

We again proceed in two steps. In the first step, we apply partykit and score-guided semtree forests to a single ordinal sample of simulation 2 to test whether the methods are able to detect DIF in a sample in which the subgroup structure is complex and the assumed MGR model does not hold for every individual in the sample. The data of half of that sample includes the same response variables as the initial sample of simulation 1 (i.e., except for the randomly generated data points). The partitioning variables are re-simulated. The input parameters are shown in Supplemental Material S1 in Table 1 and 2 in column *R*_1_ and *R*_2_. For every computed decision tree, we refit the models in each terminal node using the WLS estimator, and gather the model fit information. We compute an ensemble of 50 trees and set an RMSEA cutoff criterion of 0.05. The minimal size of the subgroups in the terminal nodes is set to 100 such that model parameters and fit indices can be estimated properly. Additionally, we set the number of variables randomly sampled as candidates at each split point to 3. For this data set, we defined the *cat*2 variable as categorical so that only two splits are necessary to retrieve the simulated subgroup *R*_1_ in a terminal node of a decision tree

$$R_{1}:=\left\{\left\{\text{cat}2\in \left\{3,4\right\}\right\}\cap \left\{\text{num}1\leq 50\right\}\right\}.$$

In the second step, we compute score-guided semtree forests for all 100 ordinal data sets and for 100 numeric data sets and analyze the method’s ability to retrieve the two simulated subgroups from a complex sample structure across multiple samples. We computed ensembles of 20 trees using the same hyperparameters as in the single sample application.

### Simulation Results

The results of the single sample application of simulation 1 are shown in Figure 1(a) (partykit), 1b (score-guided semtree), and 1c (naive semtree). When using partykit and score-guided semtree for MGR models (Figures 1(a) and (b)), all subgroups (*R*_1_ to *R*_4_) were retrieved correctly. For the naive semtree (Figure 1(c)), however, the algorithm did not stop splitting although the parameters in a terminal node are stable. These results indicate that partykit as well as score-guided semtree may be used for DIF detection in a sample that has a clear subgroup structure and for which the assumed MGR model is generally true. For the naive semtree method, on the other hand, it seems like the LR-test does not perform well with respect to numerical covariates.

When it comes to computation time, there are considerable differences between the three methods. The computation of the partykit tree took 361.5 seconds (6 minutes), the computation of score-guided semtree took 7.8 seconds, and the computation of the naive semtree algorithm took 4357 seconds (1.2 hours). These applications were conducted on a processor with a single core and 8 GB RAM. The runtime results show that naive semtree algorithm is computationally demanding and not a reasonable candidate for growing a decision tree ensemble. The modern, score-guided semtree, on the other hand, appears to be a considerably more practical method for the detection of DIF in MGR models, also in comparison to partykit. As it allows to choose from different types of score-based test statistics, semtree appears to be a good candidate to efficiently calculate robust tree ensembles.

**Figure 2.**
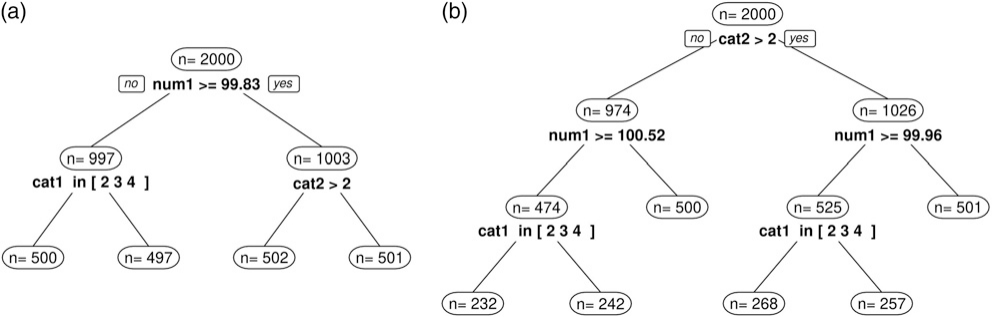
Examples for tree instability in simulation 1. (a) Inaccurate split point selection. (b) Incorrect splits performed.

We analyze and compare tree stability results of 100-fold simulations between partykit and score-guided semtree as well as between ordinal and numerical response data. We define three levels of tree stability. A stable tree is defined as a tree in which all splits have been performed at the correct split points using the correct partitioning variables and all individuals in the sample are correctly distributed among the terminal nodes. An example for such a perfect split result is shown in Figures 1(a) and (b). The second level of tree stability is defined as a tree in which the split point on the numerical variable num1 has not been perfectly detected so that not all individuals in the sample are correctly distributed among the terminal nodes. An example for such an imperfect split result is shown in Figure 2(a). The third level of tree stability is defined as a tree in which one or more faulty splits have been performed. An example for such an incorrect split result is shown in Figure 2(b).

The results of applying partykit and score-guided semtree to 100 numeric and 100 ordinal samples are shown in Table 2. The tree stability patterns show no strong differences between partykit and semtree. However, there are apparent differences when comparing the applications on ordinal and numerical response data. With numerical response data, more trees were perfectly stable. However, this is only due to a higher rate of inaccuracies in split point selection and not due to more (fully) incorrect splits with ordinal response data.

**Table 2.** Tree stability across repetitions in simulation 1.

	Ordinal data			Numerical data		
	Perfect splits	Inaccurate split point	Incorrect splits	Perfect splits	Inaccurate split point	Incorrect splits
Partykit	42%	48%	10%	76%	14%	10%
Semtree	40%	47%	13%	69%	13%	18%

The samples for simulation 2 included two subgroups with DIF (*R*_1_ and *R*_2_) and random data such that the PIEG model only holds for a portion of the sample. In addition, the simulated subgroups are not retrievable through one single decision tree. In a single sample application, we first investigate if a forest of decision trees is able to correctly detect the simulated subgroups in the data set. As shown in Tables 3 and 4, both methods are successful in the retrieval of the two subgroups as those subgroups are repeatedly identified with best model fit. However, there were other subgroups that also fit the data well (i.e., model fit estimate fell under RMSEA cutoff) although these subgroups were not explicitly simulated to fit the data. It becomes apparent that the other subgroups identified by the forests are (random) subsets of either *R*_1_ or *R*_2_. This result indicates that not all of the subgroups with acceptable model fit indices in the tree ensembles should be strictly interpreted as subgroups in which the assumed model is inherently true. Those groups with the best model fit that do not share any subset with another subgroup in the list, however, may be interpreted as subgroups in which the assumed model holds.

**Table 3.** Results of the application of semtree forest for MGR models to the sample of simulation 2. Subgroups with best model fit are shown. The column label “Freq.” refers to the number of decision trees in the forest that identified the respective subgroup.

Sim. Subgrp	Decision rule	Freq	*n*	RMSEA	*p*-value χ^2^-test
✓	$R_{semtree_{1}}:=\left\{\left\{\text{cat}2\in \left\{3,4\right\}\right\}\cap \left\{\text{num}1\leq 49.88\right\}\right\}$	3	499	0	0.592
✓	$R_{semtree_{2}}:=\left\{\left\{\text{dicho}1=0\right\}\cap \left\{\text{cat}1\in \left\{1,4,5\right\}\right\}\right\}$	1	500	0	0.568
✗	$R_{semtree_{3}}:=\left\{\left\{\text{dicho}1=1\right\}\cap \left\{\text{cat}2\in \left\{3,4\right\}\right\}\cap \left\{\text{num}1\leq 49.99\right\}\right\}$	1	254	0	0.792
✗	$R_{semtree_{4}}:=\left\{\left\{\text{cat}1\in \left\{1,4,5\right\}\right\}\cap \left\{\text{num}1\leq 49.51\right\}\cap \left\{\text{cat}2\in \left\{3,4\right\}\right\}\right\}$	4	146	0	0.462
✗	$R_{semtree_{5}}:=\left\{\left\{\text{num}1\geq 53.82\right\}\cap \left\{\text{cat}1\in \left\{1,4,5\right\}\right\}\cap \left\{\text{dicho}1=0\right\}\right\}$	7	373	0.011	0.402
✗	$R_{semtree_{6}}:=\left\{\left\{\text{cat}1\in \left\{1,4,5\right\}\right\}\cap \left\{\text{num}1\geq 49.51\right\}\cap \left\{\text{dicho}1=0\right\}\right\}$	2	382	0.011	0.398
✗	$R_{semtree_{7}}:=\left\{\left\{\text{cat}1\in \left\{1,4,5\right\}\right\}\cap \left\{\text{num}1\leq 49.98\right\}\cap \left\{\text{cat}2\in \left\{3,4\right\}\right\}\right\}$	2	147	0.016	0.411
✗	$R_{semtree_{8}}:=\left\{\left\{\text{cat}2\in \left\{1,2,5\right\}\right\}\cap \left\{\text{cat}1\in \left\{1,4,5\right\}\right\}\cap \left\{\text{dicho}1=0\right\}\right\}$	3	349	0.027	0.198
✗	$R_{semtree_{9}}:=\left\{\left\{\text{cat}1\in \left\{2,3\right\}\right\}\cap \left\{\text{num}1\leq 49.91\right\}\cap \left\{\text{cat}2\in \left\{3,4\right\}\right\}\right\}$	4	353	0.028	0.179

**Table 4.** Results of the application of partykit forest for MGR models to the sample of simulation 2. Subgroups with best model fit are shown. The column label “Freq.” refers to the number of decision trees in the forest That identified the respective subgroup.

Sim. Subgrp	Decision rule	Freq	*n*	RMSEA	*p*-value χ^2^-test
✓	$R_{mob_{1}}:=\left\{\left\{\text{cat}2\in \left\{3,4\right\}\right\}\cap \left\{\text{num}1\leq 49.87\right\}\right\}$	2	499	0	0.592
✓	$R_{mob_{2}}:=\left\{\left\{\text{dicho}1=0\right\}\cap \left\{\text{cat}1\in \left\{1,4,5\right\}\right\}\right\}$	7	500	0	0.568
✗	$R_{mob_{3}}:=\left\{\left\{\text{dicho}1=1\right\}\cap \left\{\text{cat}2\in \left\{3,4\right\}\right\}\cap \left\{\text{num}1\leq 49.39\right\}\right\}$	1	252	0	0.823
✗	$R_{mob_{4}}:=\left\{\left\{\text{cat}1\in \left\{1,4,5\right\}\right\}\cap \left\{\text{num}1\leq 49.51\right\}\cap \left\{\text{cat}2\in \left\{3,4\right\}\right\}\right\}$	2	146	0	0.462
✗	$R_{mob_{5}}:=\left\{\left\{\text{cat}1\in \left\{1,4,5\right\}\right\}\cap \left\{\text{num}1\leq 49.39\right\}\cap \left\{\text{dicho}1=1\right\}\cap \left\{\text{cat}2\in \left\{2,4\right\}\right\}\right\}$	1	102	0	0.523
✗	$R_{mob_{6}}:=\left\{\left\{\text{num}1>53.77\right\}\cap \left\{\text{cat}1\in \left\{1,4,5\right\}\right\}\cap \left\{\text{dicho}1=0\right\}\right\}$	2	373	0.011	0.402
✗	$R_{mob_{7}}:=\left\{\left\{\text{cat}1\in \left\{1,4,5\right\}\right\}\cap \left\{\text{num}1>49.39\right\}\cap \left\{\text{dicho}1=0\right\}\right\}$	4	382	0.011	0.398
✗	$R_{mob_{8}}:=\left\{\left\{\text{dicho}1=0\right\}\cap \left\{\text{cat}1\in \left\{2,3\right\}\right\}\cap \left\{\text{cat}2\in \left\{3,4\right\}\right\}\cap \left\{\text{num}1\leq 49.87\right\}\right\}$	1	246	0.022	0.321
✗	$R_{mob_{9}}:=\left\{\left\{\text{cat}2\in \left\{1,2,5\right\}\right\}\cap \left\{\text{cat}1\in \left\{1,4,5\right\}\right\}\cap \left\{\text{dicho}1=0\right\}\right\}$	2	349	0.027	0.198
✗	$R_{mob_{10}}:=\left\{\left\{\text{cat}1\in \left\{2,3\right\}\right\}\cap \left\{\text{num}1\leq 49.89\right\}\cap \left\{\text{cat}2\in \left\{3,4\right\}\right\}\right\}$	3	353	0.028	0.179
✗	$R_{mob_{11}}:=\left\{\left\{\text{cat}1\in \left\{2,3\right\}\right\}\cap \left\{\text{cat}2\in \left\{3,4\right\}\right\}\cap \left\{\text{num}1\leq 49.87\right\}\right\}$	1	352	0.029	0.164
✗	$R_{mob_{12}}:=\left\{\left\{\text{cat}1\in \left\{1,4,5\right\}\right\}\cap \left\{\text{dicho}1=1\right\}\cap \left\{\text{cat}2\in \left\{3,4\right\}\right\}\cap \left\{\text{num}1\leq 45.9\right\}\right\}$	1	133	0.045	0.184

The runtime of partykit and semtree forest depend on the number of trees of the ensemble. Thus, holding the number of trees constant, semtree forest take considerably less time to compute than partykit forest. In simulation 2, the computation time of the single trees in the ensembles were on average comparable to the computation times in simulation 1, as some trees grew deeper and others stopped splitting at the root node. Note that growing a forest can be parallelized and therefore the computation time of recursive partitioning forests also depends on the number of available processing cores.

Repeating the application of semtree forests with 20 trees in each ensemble on 100 ordinal data sets resulted in 95% of the forests recovering at least one simulated subgroup (*R*_1_ or *R*_2_). Furthermore, 41% of the forests recovered both *R*_1_ and *R*_2_. The same application on 100 numeric data sets resulted in 78% of the forests recovering at least one simulated subgroup and only 18% recovering both subgroups. Thus, the problem of inaccurate selection of split points in the decision tree for ordinal data seems to be solved by using partitioning tree ensembles.

## Discussion

Heterogeneity in survey samples is a common challenge when latent variable models are used to measure latent traits in substantive research. Survey data may include multiple, complex subgroups which can be subject to differential item functioning, and/or for which the implied measurement model does not hold altogether. Following the work of Strobl et al. (2015) and Komboz et al. (2018), we investigate several approaches for accounting for DIF in the most prominent type of multidimensional polytomous IRT model: the multidimensional graded response (MGR) model. By focusing on ordinal response scales and allowing for multiple latent variables, recursive partitioning for MGR models aims to tackle DIF in modeling contexts that are common in social scientific survey settings. We draw on three different recursive partitioning algorithms: naive and score-guided semtree (Arnold et al., 2021; Brandmaier et al., 2013) as well as partykit (Zeileis et al., 2008). As we utilize limited information estimation in building decision trees, we also propose practicable multi-tree extensions of partykit and semtree for MGR models. These approaches allow to account for instabilities in the tree growing process while maintaining computational feasibility.

In simulation 1, we demonstrated that partykit and score-guided semtree can be used to correctly find subgroups with DIF in MGR models. Comparing the algorithms using data in which the assumptions underlying ML estimation are compromised (i.e., ordinal response data) versus data in which these assumptions are not compromised (i.e., numeric response data) showed that there are not more incorrect splits performed with ordinal data. The results of the simulation study performed in Supplemental Material S2 support this finding as they indicate that different strucchange tests used on ordinal data do not perform worse than the same tests used on numeric data. However, compromising the MGR model assumptions during the tree growing process can lead to more inaccurate split points, at least for numerical partitioning variables.

The results of simulation 2 showed that a forest of semtrees is computationally more practical than a forest of partykit trees. The repeated application of semtree ensembles indicated that it is possible to retrieve subgroups with DIF from data with complex subgroup structures using tailored tree ensemble approaches. Our simulation also showed that applying such a tree ensemble method to numeric response data does not lead to better subgroup recovery. This result suggests that an ensemble method may be able to account for the instabilities of the tree caused by the compromised MGR model assumptions during tree growth. Note that in real applications, samples consist of complex subgroup structures anyway, and tree instability may be present even if the assumptions of the underlying model are not compromised. We may thus conclude that partykit and (ensembles of) semtree for MGR models represent useful tools for researchers working with multidimensional latent variable models and ordinal items in survey data.

Note that in extending recursive partitioning for MGR models to a tree ensemble method, we do no longer focus exclusively on detecting DIF. We rather consider the possibility that the assumed model structure underlying the ordinal items does not hold for all subgroups of the sample. Additionally, we acknowledge that the subgroup structure may be too complex to be disentangled by a single decision tree. In other words, an ensemble of recursive partitioning trees for MGR models recognizes that traditional data models, such as MGR models, are often not complex enough to accurately represent the internal processes of all respondents in deciding which categories to check off on survey scale items. It is rather likely that the assumption of a fixed model structure with stable parameters does not hold for every individual in every context. In these cases, parameter heterogeneity and model fit heterogeneity can be expected.

For this reason, we use a hybrid approach that includes an algorithmic model (random forest) and a data model (multidimensional GRM). Methods from the algorithmic modeling culture assume that natural mechanisms that produce data are unknown. Algorithmic models are usually used as “black boxes” to predict outcomes of such natural mechanisms (Breiman, 2001b, p. 205). Models from the data modeling culture, on the other hand, are typically restrictive explanatory models used to estimate parameters that are then used to test causal explanations. Algorithmic models need to be flexible enough to approximate the data generating mechanism well while also being robust to changes in the data. This compromise is referred to as the bias-variance trade-off in the algorithmic modeling literature (Hastie et al., 2009, p. 37). A recursive partitioning ensemble reduces bias by identifying various decision rules and associated parameter values for which the assumed model fits. It is these decision rules that lead to conditions under which controlling for DIF in MGR models actually reduces bias. Variance in tree ensembles for MGR models, on the other hand, can be controlled via the minimum size of the subgroups in the terminal nodes. Further extensions to this end could include the use of bootstrap resampling in tree ensembles for MGR models.

## Supplemental Material

Supplemental Material - Detecting Differential Item Functioning in Multidimensional Graded Response Models With Recursive Partitioning Supplemental Material for Detecting Differential Item Functioning in Multidimensional Graded Response Models With Recursive Partitioning by Franz Classe, and Christoph Kern in Applied Psychological Measurement.
