# Supplementary material for: Detecting Differential Item Functioning in Multidimensional Graded Response Models With Recursive Partitioning
Source: Appl Psychol Meas. 2024 Mar 13;48(3):83–103. doi: 10.1177/01466216241238743 (PMC10993862; doi:10.1177/01466216241238743)
Supplement: Supplemental Material - Detecting Differential Item Functioning in Multidimensional Graded Response Models With Recursive Partitioning [file sj-pdf-1-apm-10.1177_01466216241238743.pdf]

Supplementary Material

November 13, 2023

S1 Additional Tables and Figures

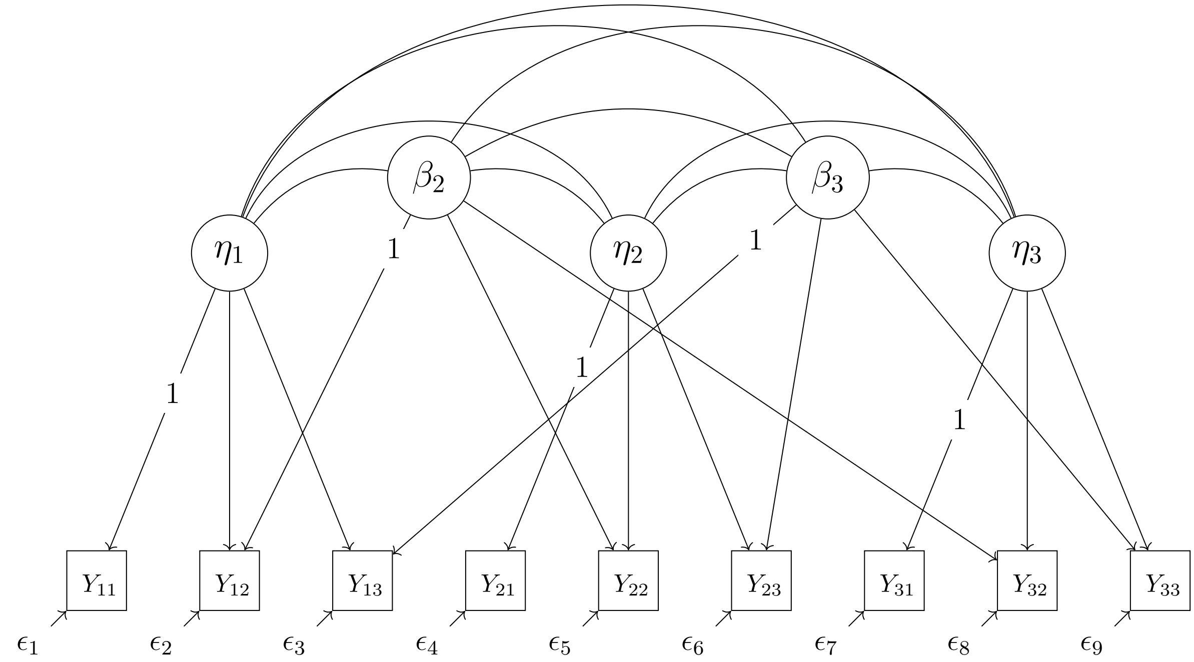

Figure 1: PIEG model for three time points t and three items i. One latent state variable η_t is assumed for each time point. Item 1 serves as reference item so that β_2 and β_3 are the only latent item effect variables in the model.

Table 1: Input variances and covariances for simulation 1 (R_1 to R_4) and simulation 2 (R_1 and R_2).

Parameter	Subgroup			
	R_1	R_2	R_3	R_4
$Var(\eta_1)$	0.27	0.37	0.51	0.47
$Var(\eta_2)$	0.27	0.21	0.30	0.28
$Var(\eta_3)$	0.27	0.34	0.42	0.24
$Var(\beta_2)$	0.34	0.49	0.44	0.39
$Var(\beta_3)$	0.22	0.31	0.39	0.25
$Cov(\eta_1, \eta_2)$	0.10	−0.05	0.15	0.26
$Cov(\eta_1, \eta_3)$	0.06	−0.03	0.11	−0.14
$Cov(\eta_2, \eta_3)$	−0.08	0.12	0.11	0.04
$Cov(\eta_1, \beta_2)$	−0.06	−0.09	0.03	−0.33
$Cov(\eta_1, \beta_3)$	0.07	0.09	0.10	−0.17
$Cov(\eta_2, \beta_2)$	−0.21	−0.05	0.03	−0.10
$Cov(\eta_2, \beta_3)$	−0.12	−0.03	0.14	−0.10
$Cov(\eta_3, \beta_2)$	0.06	0.21	−0.29	0.12
$Cov(\eta_3, \beta_3)$	0.05	0.12	−0.17	0.04
$Cov(\beta_2, \beta_3)$	0.09	0.19	0.06	−0.01

Table 2: Input threshold parameters for simulation 1 (R_1 to R_4) and simulation 2 (R_1 and R_2).

Parameter		Subgroup			
		R_1	R_2	R_2	R_2
Y_{11}	κ_{111}	−0.57	−1.16	−1.21	0.19
	κ_{112}	−0.14	−0.53	−0.58	0.81
	κ_{113}	0.27	−0.06	0.02	1.30
	κ_{114}	0.73	0.42	0.69	1.89
Y_{12}	κ_{121}	−1.18	−0.22	−0.96	−0.01
	κ_{122}	−0.47	0.49	0.02	0.39
	κ_{123}	0.07	1.16	0.86	0.72
	κ_{124}	0.67	1.88	1.72	1.14
Y_{13}	κ_{131}	−0.65	−1.43	−1.83	−1.23
	κ_{132}	0.14	−0.49	−0.81	−0.68
	κ_{133}	0.74	0.24	0.01	−0.19
	κ_{134}	1.51	1.00	0.96	0.33
Y_{21}	κ_{211}	−2.02	0.20	−0.19	0.16
	κ_{212}	−1.56	0.58	0.33	0.61
	κ_{213}	−1.14	0.94	0.74	0.97
	κ_{214}	−0.69	1.36	1.24	1.46
Y_{22}	κ_{221}	−0.61	−0.47	−2.25	0.19
	κ_{222}	−0.23	0.14	−1.49	0.75
	κ_{223}	0.12	0.80	−0.81	1.33
	κ_{224}	0.58	1.42	−0.02	1.88
Y_{23}	κ_{231}	−2.29	−1.83	0.32	−1.34
	κ_{232}	−1.80	−1.26	1.15	−0.76
	κ_{233}	−1.39	−0.72	1.93	−0.35
	κ_{234}	−0.96	−0.12	2.83	0.19
Y_{31}	κ_{311}	−0.46	−0.51	−0.93	0.42
	κ_{312}	0.01	−0.01	−0.39	0.81
	κ_{313}	0.42	0.43	0.13	1.20
	κ_{314}	0.84	0.96	0.68	1.61
Y_{32}	κ_{321}	−1.45	−1.73	−0.73	−1.34
	κ_{322}	−0.67	−0.75	−0.26	−0.59
	κ_{323}	−0.05	0.07	0.12	0.15
	κ_{324}	0.72	1.14	0.58	1.02
Y_{33}	κ_{331}	−0.67	−1.09	−1.58	−1.15
	κ_{332}	0.04	−0.21	−1.06	−0.44
	κ_{333}	0.67	0.51	−0.49	0.16
	κ_{334}	1.33	1.32	0.14	0.81

Algorithm 1: Naive `semtree` for MGR models

Initialization: Assign data to root node
Parameters: minimum sample size in terminal node, p-value threshold

```
Estimate model parameters in θ for the sample in the current node using the ML
  estimator (template model);
Compute augmented models for all possible split points for all partitioning
  variables;
Compute log-likelihood ratio of all augmented models against template model;
Set optimal split point for every partitioning variable;
Perform LR test for every partitioning variable;
if minimum p-value exceeds threshold OR min node size reached then
    end partitioning;
else
    select partitioning variable with lowest p-value in LR test;
    split node into two subnodes at optimal split point;
    for each node of current tree do
        continue partitioning process;
    end
end
for each terminal node do
    re-fit models using WLS estimator;
end
```

Algorithm 2: `partykit` for MGR models

Initialization: Assign data to root node
Parameters: minimum sample size in terminal node, p-value threshold

Estimate model parameters in $\boldsymbol{\theta}$ for the current node using ML estimation;
Assess item parameter instability though generalized M-fluctuation test with respect to each covariate $Z_1, \ldots, Z_R$;
if *minimum p-value exceeds threshold OR min node size reached* **then**
    end partitioning;
else
    detect covariate Z_{r^*} with the strongest instability;
    select the unique value as split point that **maximizes the sum of the objective functions of the two segmentations**;
    split node into two subnodes at split point;
    for *each node of current tree* **do**
        continue partitioning process;
    end
end
for *each terminal node* **do**
    re-fit models using WLS estimator;
end

Algorithm 3: Score-guided `semtree` for MGR models

Initialization: Assign data to root node

Parameters: minimum sample size in terminal node, p-value threshold

```
Estimate model parameters in θ for the current node using ML estimation;
Assess item parameter instability though generalized M-fluctuation test with
  respect to each covariate Z_1, ..., Z_R;
if minimum p-value exceeds threshold OR min node size reached then
    end partitioning;
else
    detect covariate Z_{r*} with the strongest instability;
    select the unique value as split point that maximizes the score-based test
      statistic;
    split node into two subnodes at split point;
    for each node of current tree do
        continue partitioning process;
    end
end
for each terminal node do
    re-fit models using WLS estimator;
end
```

Algorithm 4: Recursive partitioning forest for MGR models

Parameters: minimum sample size in terminal node, M-fluctuation test p-value cutoff, number of trees B, partitioning variable subset size, χ^2-test p-value or RMSEA cutoff

```
for b = 1 to B do
    Grow recursive partitioning tree using partykit or semtree for MGR with
      random draws from partitioning variables;
    save decision rules and model fit indices for terminal nodes;
end
Select exclusive subgroups with model fit indices that don't exceed cutoff;
```

S2 Performance of the Generalized M-fluctuation Test with Ordinal Data

We generate multiple samples to test the performance of the generalized M-fluctuation test with numerical and ordinal data. `partykit` and `semtree` use the results of the generalized M-fluctuation test to decide if the sample should be split into groups. The results of the test also guide the selection of the partitioning variable Z_{r^*}. `semtree` even uses the test statistic associated with the M-fluctuation test to determine the split point. The M-fluctuation test, in turn, draws on the scores of the fitted model. It is thus crucial for `partykit` and for `semtree` that the M-fluctuation test detects parameter stability correctly, even if parameter estimates derived from ordinal data are based on model assumptions of a common CFA model for metric items.

We simulate samples with $250, 500, 750$ and 1000 observations with numerical response variables for which a model holds that has the same structure as the outlined PIEG model. Furthermore, corresponding samples with ordinal response variables are simulated for which the PIEG model is true. The parameters are stable for all simulated observations, i.e., there is no DIF. All samples are based on the same input parameters for latent variable variances, latent variable covariances, and mean structure. For the ordinal data set, 36 input threshold parameters are created instead of input intercepts. For both types of response variables (numerical and ordinal) and all four sample sizes $(250, 500, 750, 1000)$, we repeat the sampling process 1000 times to compile the final set of simulated data sets.

Next, the common CFA model for numerical data (with 33 parameters) is fitted using the ML estimator to all data sets and the generalized M-fluctuation test is applied, using one random numerical and one random categorical partitioning variable. We use all six test statistics that are offered in the `semtree` R-package (Arnold et al., 2021) to compute the result of the generalized M-fluctuation test. This includes three test statistics for the numerical covariate and three test statistics for the categorical covariate (see Merkle & Zeileis, 2013; Merkle et al., 2014). With this setup, we can determine how the generalized M-fluctuation test performs when the assumptions of a common CFA model are tested

with data that follows a MGR model, and which test statistics are least susceptible to this type of misspecification.

Results. The simulation results are shown in Table 3. We calculated the percentage across all simulated data sets for which the generalized M-fluctuation test is significant (p-value below 0.05). Because the parameters in the simulated samples are stable, we denote this number as the dropout rate. Notably, none of the generalized M-fluctuation tests for models fitted to simulated numerical response variables performed considerably better than the tests for models fitted to simulated ordinal response variables. Even for small sample sizes, there is no test statistic that yields larger dropout rates for simulated ordinal responses. With numeric covariates, the CvM test statistic yields very high dropout rates of around 50% for both ordinal and numeric response variables. However, this is due to the fact that critical values of the CvM statistic are not provided for models with more than 25 parameters. The best tests statistics for multivariate latent variable models with a considerable amount of parameters (33 in our simulation) seem to be the maxLM and DM statistic for numerical covariates (see Merkle & Zeileis, 2013) and the LM statistic for categorical covariates (see Merkle et al., 2014).

Table 3: Results of simulation. The proportion of p-values of the generalized M-fluctuation test across simulated data sets that are smaller than 0.05 are shown. The column label 'numerical' indicates that numerical response variables were simulated, the label 'ordinal' indicates that ordinal response variables were simulated.

Numerical covariate

	DM		CvM		maxLM	
n	numerical	ordinal	numerical	ordinal	numerical	ordinal
250	2.3%	2.2%	50.3%	48.2%	1.9%	2.0%
500	3.8%	4.5%	45.3%	46.0%	4.2%	3.1%
750	4.5%	3.6%	46.5%	45.9%	4.6%	3.1%
1000	5.2%	3.5%	49.4%	46.4%	4.6%	4.1%

Categorical covariate

	LM		WDM		maxLM_O	
n	numerical	ordinal	numerical	ordinal	numerical	ordinal
250	3.4%	4.0%	6.4%	4.8%	4.8%	4.0%
500	4.6%	4.4%	7.5%	5.0%	5.7%	5.1%
750	5.8%	4.9%	5.6%	4.2%	6.2%	4.2%
1000	5.0%	3.9%	5.9%	6.3%	5.9%	5.3%

S3 Model Based Recursive Partitioning for MGR Models with Full Information Estimation

S3.1 Methodology

For a small number of items with a small number of response categories, there are a multitude of unique possible response patterns for the individual respondent. A response pattern y_r indicates a sequence of k_i, that is

$$y_r = \{k_1, k_2, \ldots, k_m\}. \tag{1}$$

For m items with l_i response categories, there are $\prod_{i=1}^{m} l_i$ different response patterns. A full information approach to estimating the parameters of the MGR model uses all the information contained in these response patterns (Forero & Maydeu-Olivares, 2009). The standard full information estimation method for MGR models is the *marginal maximum likelihood* (MML) method that is usually computed via the expectation-maximization (EM) algorithm (Bock & Aitkin, 1981).

In the MGR model, it is assumed that there is local independence, so that within a group of respondents with the same values for $\boldsymbol{\xi}$, the distributions of item responses are independent of each other (Samejima, 1997). Therefore, the $\boldsymbol{\xi}$-conditional probability of answering in response pattern y_r is

$$P(\boldsymbol{Y} = y_r \mid \boldsymbol{\xi}) = \prod_{i=1}^{m} P(Y_i = k_i \mid \boldsymbol{\xi}), \tag{2}$$

For a random subject sampled from a population with a continuous multivariate ability distribution $g(\boldsymbol{\xi})$, the unconditional probability of answering in response pattern y_r is

$$P(\boldsymbol{Y} = y_r) = \int_{-\infty}^{\infty} P(Y = y_r \mid \boldsymbol{\xi}) g(\boldsymbol{\xi}) \ d\boldsymbol{\xi}, \tag{3}$$

where $\int$ is a p-dimensional multiple integral. The EM algorithm estimates the probability $P(Y = y_r)$ at every iteration through numerical approximation of the p-dimensional

integral. A disadvantage of this approach is the considerable amount of computing power required. The computational burden increases exponentially with an increasing number of latent variable dimensions (Forero & Maydeu-Olivares, 2009).

The MML method is used to find the best estimates for the item parameters in $\boldsymbol{\vartheta}$ (see Equation 2) that maximize the probability for all respondents to answer in their respective response patterns. Maximizing the log likelihood is equivalent to minimizing the objective function $F_{MML}(\boldsymbol{\vartheta})$ through the EM algorithm. Let n be the sample size and $p_r = n_r/n$ be the relative frequency of occurrence of response pattern y_r. In a sense, the objective function represents the difference between the relative frequency p_r of a certain response pattern y_r and the unconditional probability of answering in that response pattern, that is

$$F_{MML}(\boldsymbol{\vartheta}) = \sum_r p_r[\ln p_r - \ln P(Y = y_r)]. \tag{4}$$

The minimalization algorithm generates successive parameter estimations $\boldsymbol{\vartheta}^{(1)}, \boldsymbol{\vartheta}^{(2)}, \ldots,$ such that

$$F_{MML}[\boldsymbol{\vartheta}^{(s+1)}] < F_{MML}[\boldsymbol{\vartheta}^{(s)}]. \tag{5}$$

At every other iteration of the minimization algorithm, the gradient of the objective function is used as the search direction (gradient descent approach). This way, the next set of parameter estimates can be chosen so that the objective function $F_{MML}(\boldsymbol{\vartheta})$ decreases (Jöreskog & Moustaki, 2006).

When the objective function F_{MML} is used, the overall model fit can be tested for by using the test statistic $T_{MML} = 2NF_{MML}(\boldsymbol{\vartheta})$. Thus, T_{MML} is $2N$ times the minimum value of the fit function $F_{MML}(\boldsymbol{\vartheta})$. The test statistic T_{MML} is asymptotically χ^2 distributed with degrees of freedom equal to the number of different response patterns minus one minus the number of independent elements of $\boldsymbol{\vartheta}$ (Jöreskog & Moustaki, 2006). It can then be used to test the model against the associated saturated model in which all possible parameters are freely estimated. This way, a test statistic for global model fit is obtained.

Schneider et al. (2021) show that it is possible to perform the generalized M-fluctuation

test for several multidimensional polytomous IRT models that are fitted using MML estimation. We can thus perform `partykit` for MGR models while using MML estimation for growing the decision tree. We call this approach *GRM Tree*. The steps performed by GRM Tree are shown in Algorithm 5.

Algorithm 5: `partykit` for MGR models using MML estimation (GRM Tree)

Initialization: Assign data to root node
Parameters: minimum sample size in terminal node, p-value threshold

Estimate model parameters in $\boldsymbol{\vartheta}$ for the current node using MML estimation;
Assess item parameter instability though generalized M-fluctuation test with respect to each covariate $Z_1, \ldots, Z_R$;
if *minimum p-value exceeds threshold OR min node size reached* **then**
    end partitioning;
else
    detect covariate Z_{r^*} with the strongest instability;
    select the unique value as split point that maximizes the sum of the objective functions of the two segmentations;
    split node into two subnodes at split point;
    for *each node of current tree* **do**
        continue partitioning process;
    end
end

S3.2 Simulations

Measurement model. The computational requirements of MML estimation for MGR models are particularly high when the model's latent variables are correlated (Forero & Maydeu-Olivares, 2009). This is the case for the original measurement model defined in Section 3.1. In order to apply GRM Tree to the PIEG model, it must be redefined as a MGR model with orthogonal latent variables. We thus define an orthogonal PIEG model and fix the covariances of the latent variables at 0 and the variances at 1. Discrimination parameters are freely estimated.

As in Section 3.1, we consider three reference latent state variables η_t and two latent item effect variables β_i. The latent variables are derived from three items at three time points resulting in nine five-category ordinal response variables Y_{it}. The cumulative

category response function of the orthogonal PIEG model is

$$P(Y_i \geq k_i \mid \eta_t, \beta_i) = \Phi(\lambda_{it}\eta_t + \delta_{it}\beta_i - \kappa_{ikt}),$$
$$\forall\ k = 1, \ldots, 4,\ \forall\ i = 2, \ldots, 3,\ \forall\ t = 1, \ldots, 3. \tag{6}$$

In this model, there are 36 free threshold parameters (4 for every five-category item) and 15 free discrimination parameters, resulting in 51 free parameters in total. The model structure is shown in Figure 2.

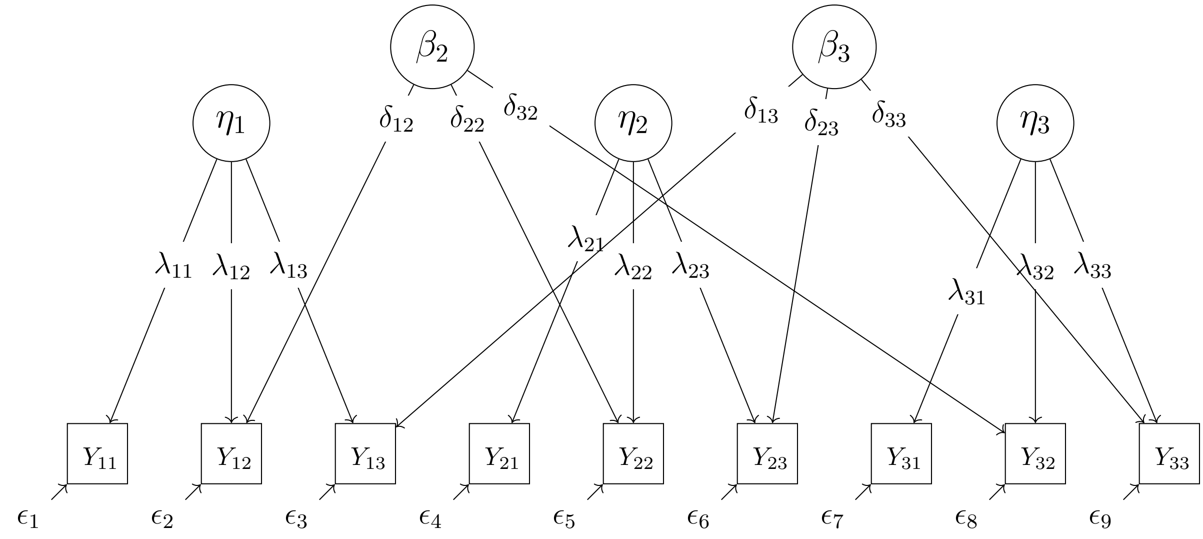

Figure 2: PIEG model with orthogonal latent variables for three time points t and three items i. One latent state variable η_t is assumed for each time point. Item 1 serves as reference item so that β_2 and β_3 are the only latent item effect variables in the model. All latent variable variances are fixed at 1.

Simulation Setup. To test GRM Tree, we simulate a sample with a similar subgroup structure as the sample of simulation 1 in Section 3.2. The only difference with respect to the subgroup structure is that the numeric partitioning variable `num1` is replaced by the categorical partitioning variable `cat3`. The structure of the entire simulated sample

can be broken down by a single decision tree. The simulated subgroups are defined as

$$R_1 \coloneqq \{\{\texttt{cat3} \in \{1,3\}\} \cap \{\texttt{cat1} \in \{1,5\}\}\},$$
$$R_2 \coloneqq \{\{\texttt{cat3} \in \{1,3\}\} \cap \{\texttt{cat1} \in \{2,3,4\}\}\},$$
$$R_3 \coloneqq \{\{\texttt{cat3} \in \{2,4,5\}\} \cap \{\texttt{cat2} \in \{1,2\}\}\},$$
$$R_4 \coloneqq \{\{\texttt{cat3} \in \{2,4,5\}\} \cap \{\texttt{cat2} \in \{3,4\}\}\}.$$

Each subgroup consists of 250 observations, and thus the full sample size is 1000. The minimum size of the terminal nodes in GRM Tree is set to 100. The outlined changes in comparison to the setup in Section 3.2 (regarding subgroup structure, sample size, and minimum terminal node size) were conducted in order to reduce the computational burden in the application of GRM Tree.

Simulation Results. The results of the GRM Tree application are shown in Figure 3. It becomes apparent that the algorithm does not retrieve the simulated subgroups correctly. The partitioning variable `cat3` is (wrongly) chosen as partitioning variable at the tree's inner nodes 2 and 7. This indicates that the results from the generalized M-fluctuation test may not be as accurate when all threshold parameters are part of the score function (see Equation 10).

An additional disadvantage of GRM Tree, compared to `partykit` or score-based SEMTree for MGR models, is the immense computation cost. The computation GRM Tree for the simulated sample described above took 450 minutes (7.5 hours) on a processor with a single core and 170GB RAM. Considering that many limitations were imposed on this particular simulation to keep computation time low, we may conclude that GRM Tree proved to be computationally impractical.

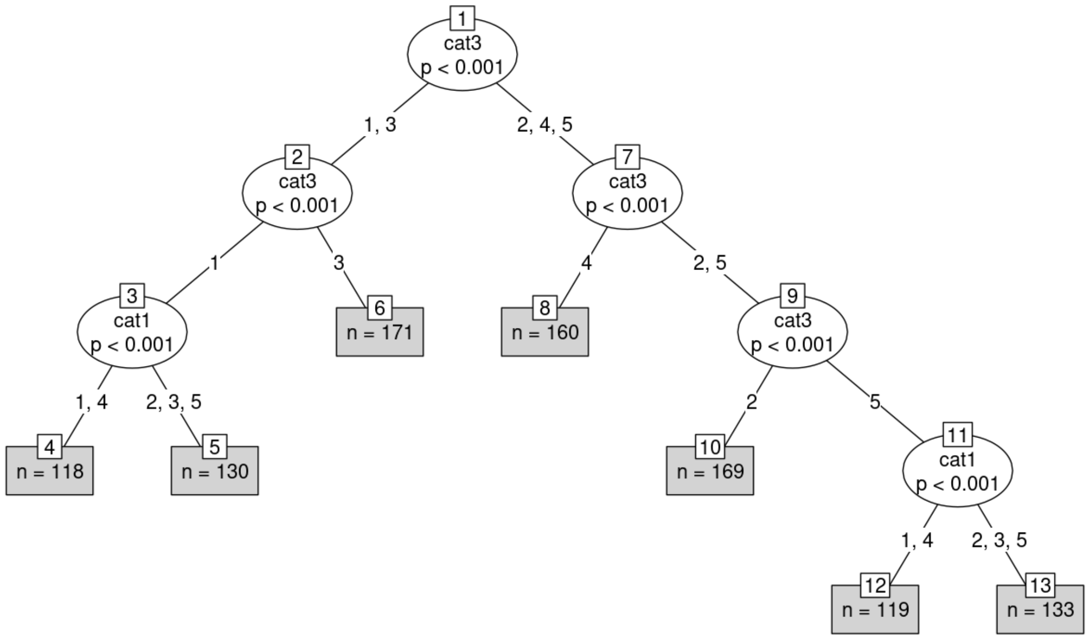

Figure 3: Results of the application of GRM Tree to simulated data.

References

Arnold, M., Voelkle, M. C., & Brandmaier, A. M. (2021). Score-guided structural equation model trees. *Frontiers in Psychology*, *11*, 564403.

Bock, R. D., & Aitkin, M. (1981). Marginal maximum likelihood estimation of item parameters: Application of an EM algorithm. *Psychometrika*, *46*(4), 443–459.

Forero, C. G., & Maydeu-Olivares, A. (2009). Estimation of IRT graded response models: limited versus full information methods. *Psychological methods*, *14*(3), 275.

Jöreskog, K. G., & Moustaki, I. (2006). Factor analysis of ordinal variables with full information maximum likelihood. *unpublished report*.

Merkle, E. C., Fan, J., & Zeileis, A. (2014). Testing for measurement invariance with respect to an ordinal variable. *Psychometrika*, *79*(4), 569–584.

Merkle, E. C., & Zeileis, A. (2013). Tests of measurement invariance without subgroups: A generalization of classical methods. *Psychometrika*, *78*(1), 59–82.

Samejima, F. (1997). Graded response model. In *Handbook of modern item response theory* (pp. 85–100). Springer.

Schneider, L., Strobl, C., Zeileis, A., & Debelak, R. (2021). An R toolbox for score-based measurement invariance tests in irt models. *Behavior Research Methods*, 1–13.
